# Environmental Correlates of H5N2 Low Pathogenicity Avian Influenza Outbreak Heterogeneity in Domestic Poultry in Italy

**DOI:** 10.1371/journal.pone.0086788

**Published:** 2014-01-22

**Authors:** Lapo Mughini-Gras, Lebana Bonfanti, Paolo Mulatti, Isabella Monne, Vittorio Guberti, Paolo Cordioli, Stefano Marangon

**Affiliations:** 1 Istituto Zooprofilattico Sperimentale delle Venezie (IZSVe), Legnaro, Padua, Italy; 2 Institute for Environmental Protection and Research (ISPRA), Ozzano dell’Emilia, Bologna, Italy; 3 Istituto Zooprofilattico Sperimentale della Lombardia e dell’Emilia Romagna (IZLER), Brescia, Italy; The University of Hong Kong, China

## Abstract

Italy has experienced recurrent incursions of H5N2 avian influenza (AI) viruses in different geographical areas and varying sectors of the domestic poultry industry. Considering outbreak heterogeneity rather than treating all outbreaks of low pathogenicity AI (LPAI) viruses equally is important given their interactions with the environment and potential to spread, evolve and increase pathogenicity. This study aims at identifying potential environmental drivers of H5N2 LPAI outbreak occurrence in time, space and poultry populations. Thirty-four environmental variables were tested for association with the characteristics of 27 H5N2 LPAI outbreaks (i.e. time, place, flock type, number and species of birds affected) occurred among domestic poultry flocks in Italy in 2010–2012. This was done by applying a recently proposed analytical approach based on a combined non-metric multidimensional scaling, clustering and regression analysis. Results indicated that the pattern of (dis)similarities among the outbreaks entailed an underlying structure that may be the outcome of large-scale, environmental interactions in ecological dimension. Increased densities of poultry breeders, and increased land coverage by industrial, commercial and transport units were associated with increased heterogeneity in outbreak characteristics. In areas with high breeder densities and with many infrastructures, outbreaks affected mainly industrial turkey/layer flocks. Outbreaks affecting ornamental, commercial and rural multi-species flocks occurred mainly in lowly infrastructured areas of northern Italy. Outbreaks affecting rural layer flocks occurred mainly in areas with low breeder densities in south-central Italy. In savannah-like environments, outbreaks affected mainly commercial flocks of galliformes. Suggestive evidence that ecological ordination makes sense genetically was also provided, as virus strains showing high genetic similarity clustered into ecologically similar outbreaks. Findings were informed by hypotheses about how ecological interactions among poultry populations, viruses and their environments can be related to the observed patterns of H5N2 LPAI occurrence. This may prove useful in enhancing future interventions by developing site-specific, ecologically-grounded strategies.

## Introduction

Low pathogenicity avian influenza (LPAI) viruses of H5 and H7 subtypes have the potential for mutation into highly pathogenic avian influenza (HPAI) virus strains [1], causing huge economic losses due to the high bird mortality rates and costs of control measures in domestic poultry [2], not to mention the potentially serious implications for human health [3]. This is the case of H5N2 LPAI viruses, for which there are several documented instances of increased virulence in domestic poultry [4]–[6], as well as human infection [7], [8].

Italy has experienced several incursions of avian influenza (AI) viruses of H5N2 subtype in domestic poultry. In 1997–1998, eight H5N2 HPAI outbreaks were detected in multi-species backyard and small commercial poultry flocks in the north-eastern regions of Veneto and Friuli Venetia Giulia [6]. Seven years later in 2005, 15 outbreaks of H5N2 LPAI were detected in industrial meat turkey flocks, clustered within a radius of 14 km in the northern region of Lombardy [9], causing more than € 5 million losses in both direct and consequential costs [2]. At that time, prophylactic vaccination of long-living poultry species (mainly turkeys and layers) with a bivalent H5/H7 vaccine based on the DIVA (Differentiating Infected from Vaccinated Animals) strategy was in force, according to European Commission Decision 2004/666/EC [10]. The increased resistance to the field virus challenge of vaccinated birds and the reduction of virus shedding, combined with on-farm active surveillance and strict control measures, resulted in a rapid eradication of the epidemic [9]. In 2007, a H5N2 LPAI outbreak was detected in a free-range duck and goose breeder flock in the northern region of Emilia-Romagna, but phylogenetic analyses revealed a poor relationship to the previous one isolated in Lombardy [11]. On the contrary, the HA and NA genes resulted to be highly similar (>99.8%) to a H5N2 LPAI virus isolated from mallards in the same time period and geographical area, suggesting a possible introduction from the wild reservoir [11]. Further outbreaks caused by H5N2 LPAI virus in domestic poultry in Italy were detected in 2010–2012 all over the country, indicating recurrent viral introductions and/or increased viral circulation in various sectors of the Italian poultry industry.

The reoccurrence and spread of H5N2 AI viruses in geographically distant areas and different poultry production sectors, despite significant control efforts implemented, is likely to be driven by dynamic interactions between poultry and the environment where these viruses circulate, and these interactions may vary along ecological gradients in a more or less structured way. Several environmental variables have been found to be related to the observed spatio-temporal patterns and molecular evolution of AI viruses, particularly HPAI virus strains, in many parts of the world [12]–[15]. It is therefore increasingly apparent that a disease ecology framework, which posits that disease emergence is the result of multifactorial interactions with the environment as a whole [16], may well provide insights about whether and how the observed spatio-temporal pattern of H5N2 AI outbreaks in varying sectors of the Italian poultry industry is driven by certain ecological pressures. This means to identify environmental variables associated with outbreak heterogeneity, which is defined here in epidemiological terms as the dissimilarity in outbreak characteristics such as time and place of outbreak occurrence, type of affected flocks, number and species of birds therein. Focussing on domestic poultry in Italy, this study aims at identifying potential environmental drivers of H5N2 LPAI outbreak heterogeneity, with an account of molecular and phylogenetic characteristics of those virus strains for which genetic sequence data were available. Although inferring any relationship of causation was beyond the objectives of this study, results were expected to help us in disentangling the pattern of (dis)similarities among the different H5N2 LPAI outbreaks as the outcome of possible large-scale, environmental interactions in ecological dimension.

## Materials and Methods

### Outbreak Description

**Figure 1:**
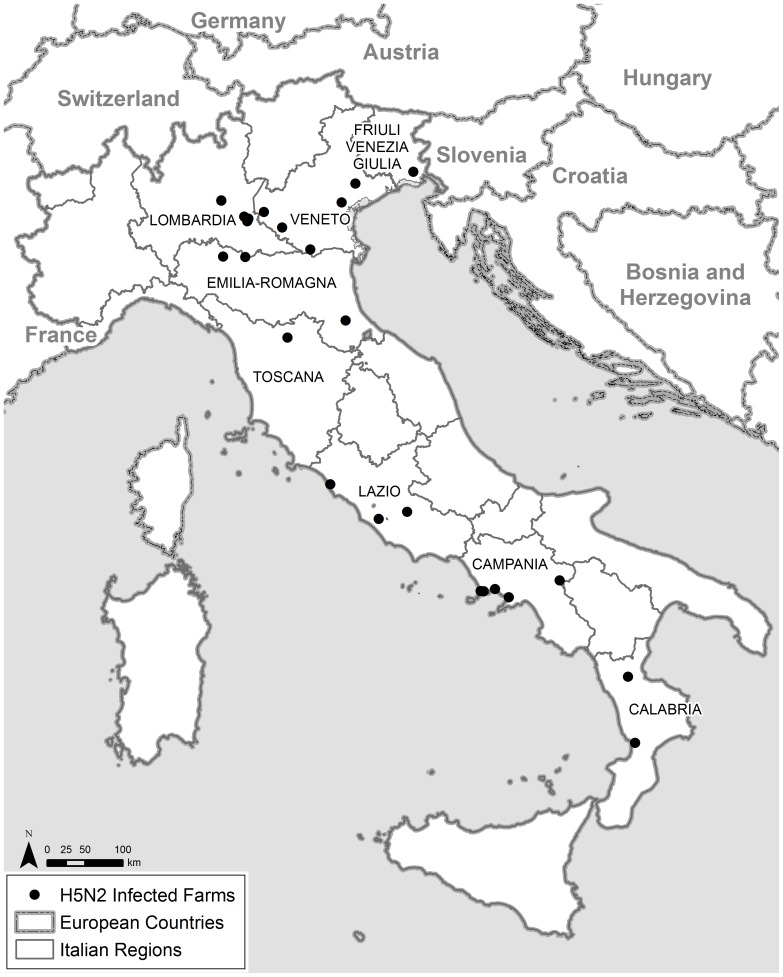
Map of the 27 Italian H5N2 LPAI outbreaks in domestic poultry, 2010–2012.

A total of 27 outbreaks of H5N2 LPAI virus, detected in domestic poultry in Italy between January 2010 and October 2012, formed the basis of this study (Table 1 and Figure 1). The first outbreak (10/1) was detected on 12 January 2010 during mandatory pre-movement testing in an multi-species ornamental poultry flock in the Veneto region. Further outbreaks were detected in backyard, ornamental, grower, dealer and industrial poultry flocks through official controls within the framework of the Italian national AI monitoring plan based on serological (hemagglutination inhibition [HI] assay in blood samples) and/or virological (real-time reverse transcriptase PCR in swab samples) testing according to European Commission Decisions 2006/437/EC and 2010/367/EC. In the seven outbreaks affecting industrial turkey flocks (11/6, 12/5, 12/6, 12/11, 12/12, 12/13 and 12/14), respiratory symptoms, reduced feed consumption and/or increased mortality were observed. All diagnoses of AI were confirmed by the National Reference Laboratory for Avian Influenza and Newcastle Disease in Legnaro (Padua), Italy.

**Table 1:** H5N2 LPAI outbreaks in Italy between 2010 and 2012.

Outbreak ID	Region	Province	Flock type	Turkeys	Broilers	Layers	Guineafowls	Pigeons	Quails	Pheasants	Ducks	Geese	Ornamental species	First sampling	HI^1^	PCR^2^
10/1	Veneto	Rovigo	Ornamental	69	534		26	622			262		142	12/01/2010	X	X
10/2	Emilia Romagna	Forlì-Cesena	Ornamental	20	188				22	18	388	21	2870	18/11/2010	X	X
10/3	Friuli Venetia Giulia	Udine	Backyard			45								02/12/2010	X	
11/1	Veneto	Verona	Backyard	8	15			15			83	38	3	14/02/2011	X	
11/2	Veneto	Venetia	Dealer		24	40	5	10			12			21/03/2011	X	X
11/3	Campania	Salerno	Grower		250									25/11/2011	X	
11/4	Campania	Avellino	Backyard			47								29/11/2011	X	
11/5	Emilia Romagna	Parma	Grower	21	271		2	45	28	18	46	9	294	30/11/2011	X	X
11/6	Latium	Viterbo	Industrial	5600										12/12/2011	X	
11/7	Calabria	Catanzaro	Backyard			30								21/12/2011	X	
11/8	Latium	Rome	Ornamental											21/12/2011	X	
11/9	Campania	Napoli	Backyard			104								28/12/2011		X
12/1	Emilia Romagna	Forlì-Cesena	Ornamental		110			13			777	36		26/01/2012		X
12/2	Campania	Napoli	Industrial			4800								03/05/2012	X	
12/3	Calabria	Cosenza	Industrial			1300								15/06/2012	X	
12/4	Campania	Napoli	Backyard			70								28/06/2012	X	
12/5	Lombardy	Brescia	Industrial	15000										31/08/2012		X
12/6	Lombardy	Brescia	Industrial	11700										03/09/2012		X
12/7	Tuscany	Prato	Backyard	1		100	21				12	34		04/09/2012		X
12/8	Lombardy	Mantua	Grower	2	5653	6			200		25			18/09/2012		X
12/9	Veneto	Treviso	Ornamental		186			100			240	128	96	24/09/2012	X	
12/10	Veneto	Verona	Grower		1950	3900	2750							28/09/2012		X
12/11	Lombardy	Mantua	Industrial	37962										05/10/2012	X	X
12/12	Lombardy	Mantua	Industrial	28268										05/10/2012	X	
12/13	Lombardy	Mantua	Industrial	14456										05/10/2012		X
12/14	Lombardy	Mantua	Industrial	16533										08/10/2012		X
12/15	Latium	Rome	Dealer			750								18/10/2012	X	

1. Diagnosis by hemagglutination inhibition assay in blood samples.

2. Diagnosis by real-time reverse transcriptase PCR in swab samples.

In 2012, following the identification of outbreak 12/5 in the Lombardy region, the Italian Ministry of Health strengthen monitoring activities in the northern regions of Veneto, Lombardy, Piedmont and Emilia-Romagna on industrial and commercial poultry flocks. In addition, fairs, markets, shows or other kinds of gathering of poultry were prohibited. Control measures were implemented by veterinary authorities according to European Council Directive 2005/94/EC, including stamping out of affected premises, cleansing and disinfection, quarantine measures and movement restrictions. In two occasions (outbreaks 10/2 and 12/9), derogation for killing ornamental bird species kept for non-commercial purposes was granted. All control measures were lifted on 21 November 2012.

Epidemiological investigations allowed veterinary authorities to detect outbreak 12/6, which affected an industrial turkey flock in the Lombardy region. Epidemiological investigation also revealed a direct connection (the same poultry company holder, workers and veterinarians, as well as very close geographical proximity) between outbreaks 12/14 and 12/12, and between outbreaks 12/12 and 12/13.

Information on coordinate locations of the affected flocks, date of first sampling, flock type (backyard, ornamental, grower, dealer or industrial), number and species of birds therein, were available for all the outbreaks.

### Environmental Variables

Table 2 shows the 34 environmental variables hypothesized to be related to the characteristics of the 27 H5N2 LPAI outbreaks under the disease ecology framework proposed by Carrel *et al*. [15]. Each of these variables was computed within a buffer radius of 10 km around each of the outbreaks. Data on the environmental variables were obtained from different sources (Table 2). Human population density (people/km^2^) was computed based on the figures provided by the Italian National Institute of Statistics (ISTAT) at the level of municipal census block for the year 2010. Densities of domestic poultry farms (farms/km^2^) and their respective populations (birds/km^2^), divided into turkey breeders, fattening turkeys, broiler breeders, fattening broilers, layers, ducks and geese, and other poultry, were calculated based on the georeferenced (lat./long.) official poultry census data for the year 2012 obtained from the Italian National Poultry Registry. Percent land coverage was calculated for each of the 15 land cover classes defined by the second classification level of the Corine Land Cover (CLC) 2006 dataset, version 13, provided by the European Environment Agency at a resolution of 100 m. Median, minimum and maximum elevation (m above sea level) were computed using the the Shuttle Radar Topography Mission (SRTM) Digital Elevation Data provided by the Consortium for Spatial Information of the Consultative Group for International Agricultural Research (CGIAR–CSI) at a resolution of 90 m. Wild waterfowl density (birds/km^2^) was obtained from the Italian Institute for Environmental Protection and Research (ISPRA) and associated with the related digitized wetlands at a scale 1∶10000, obtained from satellite (Land Sat) imagery.

**Table 2:** Environmental variables hypothesized to be related to differentiation among the H5N2 LPAI outbreaks.

Variable ID	Environmental variable	Measure computed(within the buffer)	Resolution of raw data	Referenceyear	Source
A	Human population density	People/km^2^	Municipal census blocks	2010	*
	Poultry population density				
B1	Turkey breeders	Birds/km^2^	Cartesian coordinates	2012	**
B2	Fattening turkeys	Birds/km^2^	Cartesian coordinates	2012	**
B3	Broiler breeders	Birds/km^2^	Cartesian coordinates	2012	**
B4	Fattening broilers	Birds/km^2^	Cartesian coordinates	2012	**
B5	Laying hens	Birds/km^2^	Cartesian coordinates	2012	**
B6	Ducks and geese	Birds/km^2^	Cartesian coordinates	2012	**
B7	Other poultry	Birds/km^2^	Cartesian coordinates	2012	**
	Poultry farm density				
C1	Turkey breeders	Farms/km^2^	Cartesian coordinates	2012	**
C2	Fattening turkeys	Farms/km^2^	Cartesian coordinates	2012	**
C3	Broiler breeders	Farms/km^2^	Cartesian coordinates	2012	**
C4	Fattening broilers	Farms/km^2^	Cartesian coordinates	2012	**
C5	Laying hens	Farms/km^2^	Cartesian coordinates	2012	**
C6	Ducks and geese	Farms/km^2^	Cartesian coordinates	2012	**
C7	Other poultry	Farms/km^2^	Cartesian coordinates	2012	**
	Land surface area				
D1	Urban fabric	Percent	100 m grid raster	2006	†
D2	Industrial, commercial and transport units	Percent	100 m grid raster	2006	†
D3	Mine, dump and construction sites	Percent	100 m grid raster	2006	†
D4	Artificial, non-agricultural vegetated areas	Percent	100 m grid raster	2006	†
D5	Arable land	Percent	100 m grid raster	2006	†
D6	Permanent crops	Percent	100 m grid raster	2006	†
D7	Pastures	Percent	100 m grid raster	2006	†
D8	Heterogeneous agricultural areas	Percent	100 m grid raster	2006	†
D9	Forests	Percent	100 m grid raster	2006	†
D10	Scrub and/or herbaceous vegetation associations	Percent	100 m grid raster	2006	†
D11	Open spaces with little or no vegetation	Percent	100 m grid raster	2006	†
D12	Inland wetlands	Percent	100 m grid raster	2006	†
D13	Maritime wetlands	Percent	100 m grid raster	2006	†
D14	Inland waters	Percent	100 m grid raster	2006	†
D15	Marine waters	Percent	100 m grid raster	2006	†
	Elevation				
E1	Median elevation	m above sea level	90 m grid raster	2008	‡
E2	Minimum elevation	m above sea level	90 m grid raster	2008	‡
E3	Maximum elevation	m above sea level	90 m grid raster	2008	‡
F	Wild waterfowl population density	Birds/km^2^	1∶10000 digitized shapefile	2010	§

* Italian National Institute of Statistics (ISTAT); **National Poultry Registry (BDN); †Corine Land Cover (CLC) 2006 dataset, version 13; ‡Shuttle Radar Topography Mission (SRTM), CGIAR-CSI, 2008, version 4; §Italian Institute for Environmental Protection and Research (ISPRA).

### Ordination Analysis

Non-metric multidimensional scaling (NMDS) [17] was used as ordination technique to explore the underlying pattern of (dis)similarities among the 27 H5N2 LPAI outbreaks, and to further relate this pattern to the 34 environmental variables, as proposed by Carrel *et al*. [15] for scaling H5N1 AI virus isolates in Vietnam.

NMDS was used to find a configuration for the set of *n* points represented by the 27 H5N2 LPAI outbreaks in multidimensional space such that the inter-point distances corresponded as closely as possible to the observed (dis)similarities measured in *p* elements represented by the sampling date and geographical coordinates of the outbreaks, the type of affected flocks and the avian species therein (Table 1), according to a goodness-of-fit criterion called ‘stress’, which measures the degree of distortion of the ordination with respect to the original input data [17]. Conventionally, stress values <5% indicate a good fit for the data, whereas stress values >20% give unreliable results [15], [17]. After data standardization, a *n* by *p* input matrix of all pair-wise distances was calculated using the Gower’s similarity measure for mixed data types [18]. The configuration of the points in the final ordination therefore summarized the differences in spatio-temporal and poultry population (flock-related) characteristics of the 27 H5N2 LPAI outbreaks. The optimum number of dimensions used in the NMDS was chosen as to minimize stress without compromising the utility of the ordination.

Once the 27 H5N2 LPAI outbreaks were ordinated according their spatio-temporal and flock-related characteristics, each of the 34 environmental variables was aligned in the ordination space where its correlation was maximal. The coefficient of determination (*R* ^2^ statistic) was used to identify the most important environmental variables associated with the ordination configuration.

A three-dimensional NMDS was found to be the optimal configuration for the data. Thus, the obtained three sets of NMDS dimension scores, which reflected the amount of difference in the spatio-temporal and flock-related characteristics of the outbreaks, were regressed on the environmental variables using segmented (piecewise) multiple linear regression analysis as to identify the independent environmental correlates of outbreak heterogeneity. Segmented linear regression allows for the independent variables to be partitioned into intervals in order to fit a separate regression line segment to each interval. This allowed us to jointly examine the relationships of the environmental variables with outbreak heterogeneity in the three different NMDS dimensions using the same regression model. The segment breakpoints were therefore those between the first and second dimensions, and between the second and third dimensions. A positive association indicated that the environmental variable was associated with an increased outbreak heterogeneity, whereas a negative association indicated that the environmental variable was associated with a decreased outbreak heterogeneity (i.e. increased outbreak homogeneity). Phrased differently, where a positively associated variable is more represented, outbreaks would tend to occur more unpredictably in time, space and affected poultry populations, vice versa for a negatively associated variable.

Following Carrel *et al*. [15], to limit the number of variables that the analysis will focus on, only those variables with *R* ^2^ values >0.10 were included in the initial model. Variables were then dropped if they exhibited high collinearity with other variables, as indicated by a Variance Inflate Factor (VIF) >5, with the choice of which among the collinear variables to retain in the model being made based upon improved *R* ^2^. A stepwise variable selection approach was then applied to fit a parsimonious multivariable model in which variables were significantly associated with the outcome (p<0.05) and the Akaike Information Criterion (AIC) was minimized. Also the effect of removing variables on the other variables included in the model was monitored.

### Cluster Analysis

The ordinated 27 H5N2 LPAI outbreaks were assigned into clusters using Ward’s minimum variance method [19]. The Ward’s method is an iterative clustering procedure where all clusters are initially considered as singletons and then merged stepwise based on groups that lead to the minimum increase in the total within-cluster variance. The variance of the differences in attributes within a cluster is minimized using a distance algorithm based on the sum of squares of differences in the attributes, thereby combining clusters whose merge minimizes the increase in the within-group total sum of squares error [19].

Differences in environmental variables with *R* ^2^ values >0.10 were tested among the identified clusters using Kruskal-Wallis’ test (KW) and post-hoc pair-wise comparisons based on Mann-Whitney U test (MW) with Bonferroni’s adjustment of the p-value. Environmental variables significantly associated with the subdivision of outbreaks into clusters indicated how differentiation of environmental variables corresponded to differentiation in cluster assignment.

Statistical analysis was performed using STATA 11.2 (StataCorp LP, College Station, USA) and when applicable statistical significance was set to p<0.05.

### Viral Sequence Data and Phylogenetic Analysis

The complete genome sequences of five H5N2 LPAI viruses representative of the outbreaks 10/1, 11/9, 12/5, 12/10 and 12/14 were generated according to methods described previously [20]. In addition, HA and NA genes were obtained from a virus collected from the outbreak 12/6, and the HA gene only was sequenced for viruses identified from the outbreaks 12/8. The nucleotide sequences obtained in this study are made available in the GISAID database under the following accession numbers: EPI464915–EPI464957.

Nucleotide sequence alignments were manually constructed for each gene segment and for the concatenated whole genome using the Se-Al program [21]. To infer the evolutionary relationships for each gene segment, we employed the maximum likelihood (ML) method available in the PhyML program, incorporating a GTR model of nucleotide substitution with gamma-distributed rate variation among sites (with four rate categories, Γ4) and a heuristic SPR branch-swapping search procedure [22]. A bootstrap resampling process (1000 replications) using the neighbour-joining (NJ) method and incorporating the ML substitution model defined above, was employed to assess the robustness of individual nodes of the phylogeny using PAUP* [23]. Parameter values for the GTR substitution matrix, base composition, gamma distribution of the rate variation among sites, and proportion of invariant sites (I) were estimated directly from the data using MODELTEST [24].

## Results

### Outbreak Ordination

At three dimensions, stress was minimized (3.8%), indicating that the final three-dimensional NMDS ordination represented well the scaled spatio-temporal and flock-related differences among the 27 H5N2 LPAI outbreaks. The first NMDS dimension explained 69% of the variance in the data, while the second and third dimensions explained 29% and 2% of the variance, respectively.

**Figure 2:**
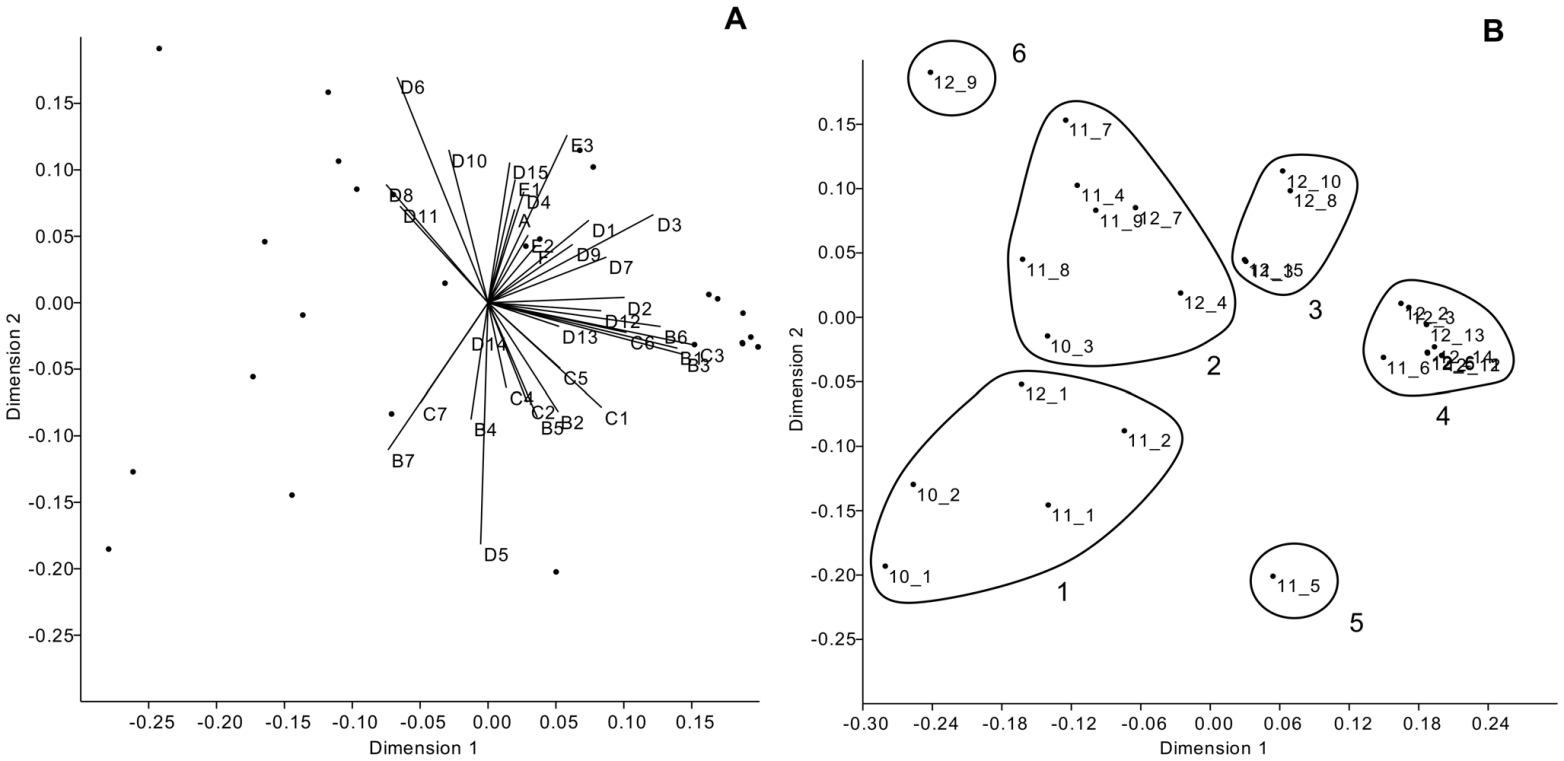
Plots of the Italian 2010–2012 H5N2 LPAI outbreaks in the first two NMDS dimensions. Plot A shows each environmental variable’s axis of differentiation, aligned in the ordination space, over the scaled spatio-temporal and flock type-related characteristics of the outbreaks. Plot B shows the final configuration of the outbreaks in the first two NMDS dimensions charted according to cluster assignment. See Tables 1 and 2 for details about the outbreaks and the environmental variables.

The first two NMDS dimensions are plotted in Figure 2 together with the 34 environmental variables hypothesized to be related to outbreak heterogeneity. Most of these variables had their axes of differentiation with the same alignment in the ordination space, mainly directed towards the higher scores of the first and second dimensions. Plotting the environmental variables onto the ordination space also indicated their differing strengths of association, with longer axes reflecting larger *R* ^2^ values.

**Figure 3:**
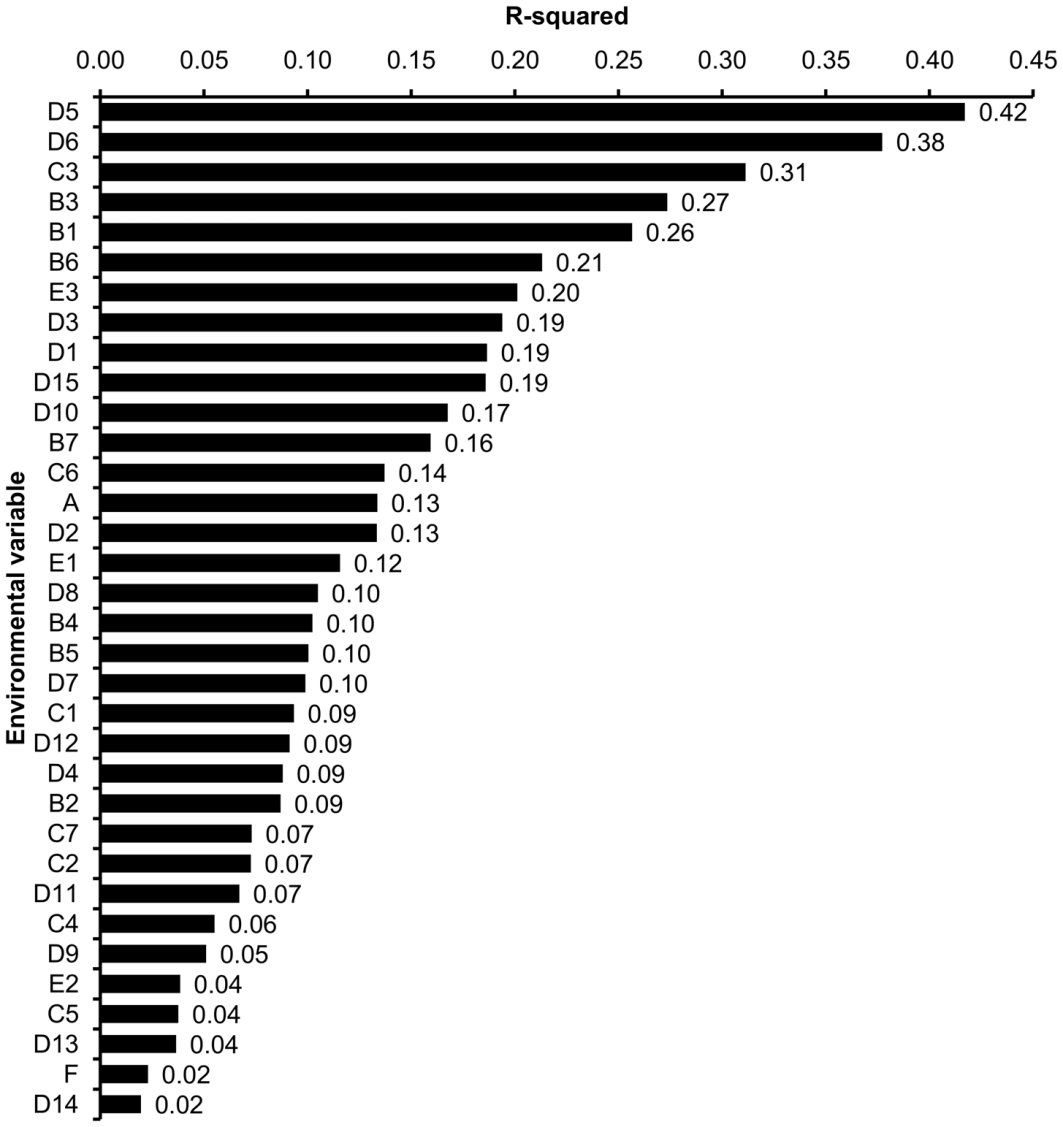
Goodness-of-fit scores (*R* ^2^) for the 34 environmental variables aligned in the ordination space. See Table 2 for details about the environmental variables.

Sixteen variables had *R* ^2^ values >0.10 (Figure 3), these were variables specifying the amounts of arable lands and permanent crops, broiler breeder farm density, broiler and turkey breeder population densities, duck and goose population density, maximum elevation, amounts of land occupied by mine, dump and construction sites, urban fabric, marine waters, scrub and/or herbaceous vegetation associations, density of poultry populations other than turkeys, layers, broilers, ducks and geese (TLBDG), duck and goose farm density, human density, industrial, commercial and transport units, and median elevation.

Of the 16 variables with *R* ^2^ values >0.10 considered for inclusion in the regression model, 10 were iteratively dropped, leading to a final model consisting of six significant predictors of outbreak heterogeneity (Table 3). Outbreak heterogeneity was positively associated with densities of broiler and turkey breeders, and with the amount of land covered by industrial, commercial and transport units. On the other hand, outbreak heterogeneity was negatively associated with the amount of arable land, and with densities of duck and goose farms, and of poultry other than TLBDG.

**Table 3:** Results from the final piecewise multiple linear regression model.

Environmental variable(associated dimension)	Coefficient(95% confidence interval)	p value
Turkey breeder population density (1^st^)	0.137 (0.058 to 0.216)	0.001
Broiler breeder population density (1^st^)	0.065 (0.028 to 0.102)	0.001
Industrial, commercial and transport units (1^st^)	0.041 (0.001 to 0.080)	0.042
Arable land (2^nd^)	−0.061 (−0.094 to −0.028)	<0.0001
Other poultry population density (2^nd^)	−0.037 (−0.070 to −0.003)	0.032
Duck and goose farm density (1^st^)	−0.113 (−0.195 to 0.031)	0.007

Coefficients, 95% confidence intervals and p values for the associations between the selected environmental variables with the three sets of NMDS dimension scores for the 27 H5N2 LPAI outbreaks.

### Cluster Assignment

The ordinated outbreaks were assigned into six clusters based upon the Ward’s method (Figure 2). Cluster 1 included mainly early outbreaks affecting ornamental, commercial and rural multi-species flocks in northern Italy; cluster 2 included mainly later outbreaks affecting rural layer flocks in south-central Italy (with the only exception of outbreak 10/3 in the north-eastern part of the country); cluster 3 included predominantly late outbreaks affecting commercial flocks with galliformes birds located throughout the country; cluster 4 included all the outbreaks affecting industrial flocks with longer-living poultry species (turkeys and layers) located throughout the country; clusters 5 and 6 consisted of only one outbreak apiece (outbreaks 11/5 and 12/9), which therefore appeared to possess the greatest ecological distances from the other outbreaks in ordination space. Among the considered outbreak characteristics, flock type was the one that determined cluster assignment more incisively, i.e. for which the within-cluster variance was minimized the most, followed by place, time and affected birds.

**Figure 4:**
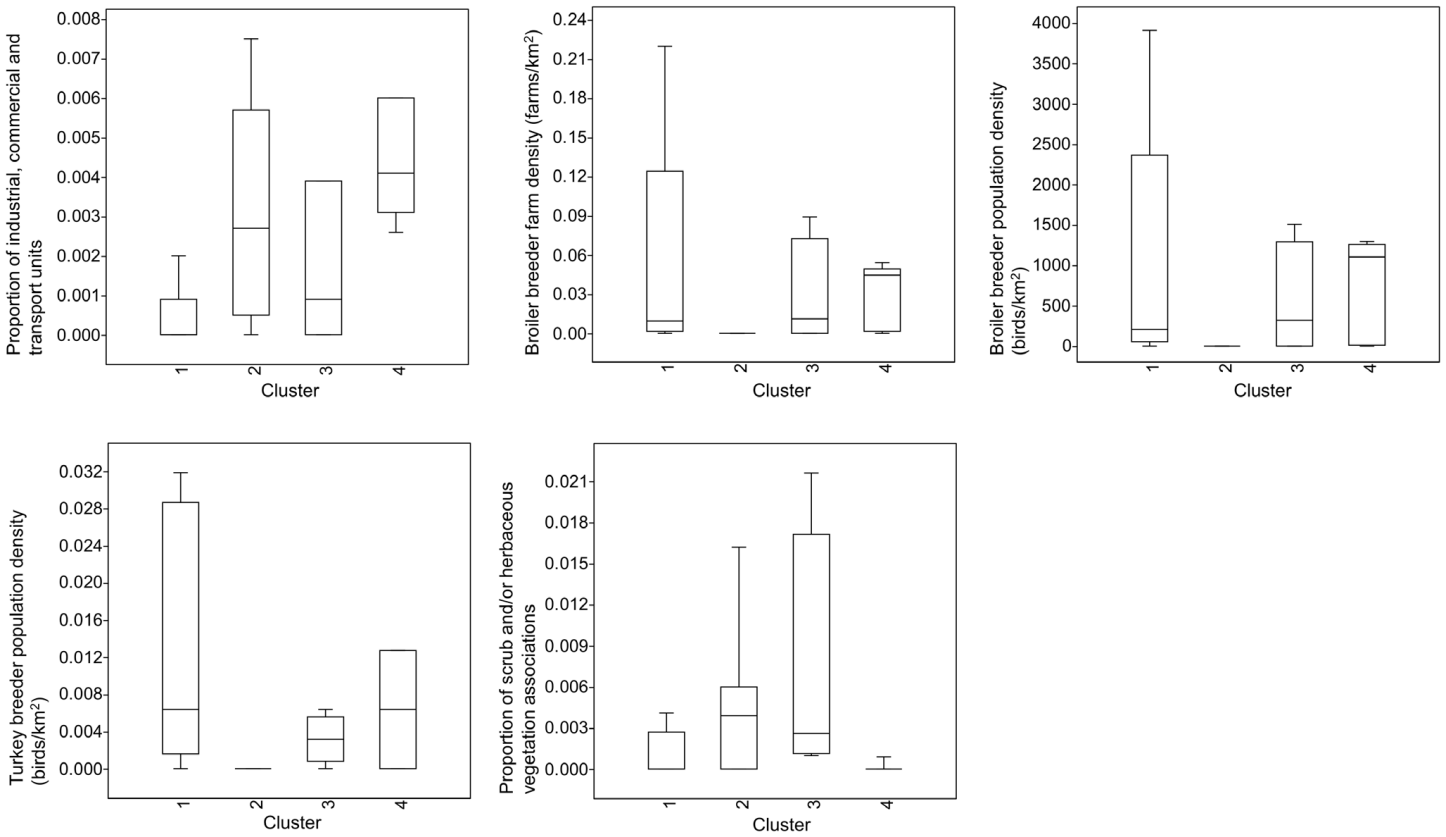
Box-plots of the five environmental variables significantly associated with the subdivision of outbreaks into clusters.

Examining the variation in the 16 environmental variables with *R* ^2^>0.10 over the four clusters containing >1 outbreak revealed that outbreaks in each of the clusters had different associations with the environmental variables. In Figure 4, box plots of the variables significantly associated with clustering are displayed, these were: 1) amount of land occupied by industrial, commercial and transport units (KW, p = 0.017), significantly higher in cluster 4 than cluster 1 (MW, adjusted p = 0.019); 2) broiler breeder farm density (KW, p = 0.027), significantly higher in cluster 4 compared to cluster 2 (MW, adjusted p = 0.030); 3) broiler breeder population density (KW, p = 0.027), significantly higher in cluster 4 compared to cluster 2 (MW, adjusted p = 0.030); 4) turkey breeder population density (KW, p = 0.018), significantly higher in cluster 4 compared to cluster 2 (MW, adjusted p = 0.048); and 5) amount of land covered by scrub and/or herbaceous vegetation associations (KW, p = 0.022), significantly higher in cluster 3 compared to cluster 4 (MW, adjusted p = 0.012).

### Molecular Characterization and Phylogenetic Analysis

**Figure 5:**
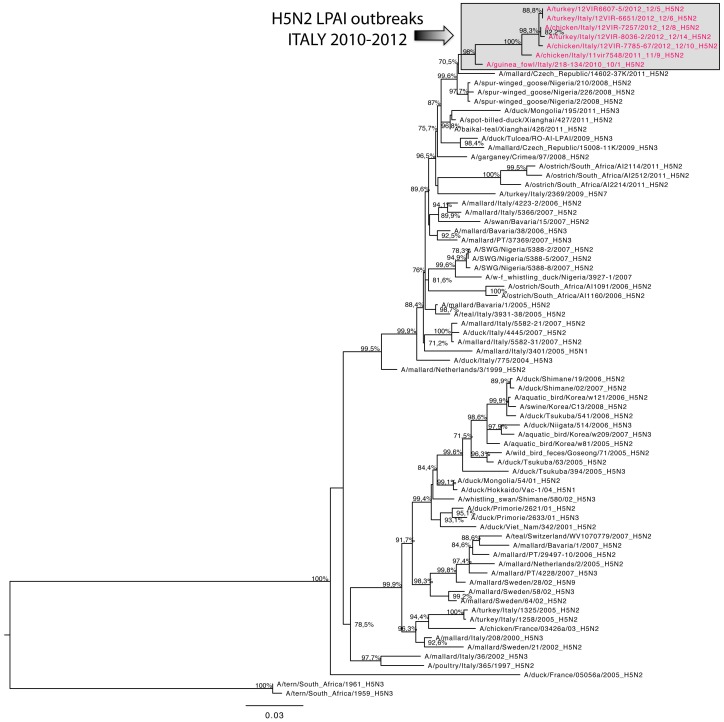
Maximum-likelihood phylogenetic tree for the hemagglutinin gene segment of Italian 2010–2012 H5N2 LPAI viruses. Viruses sequenced and characterized in this study are in red. Numbers at the nodes represent bootstrap values.

**Figure 6:**
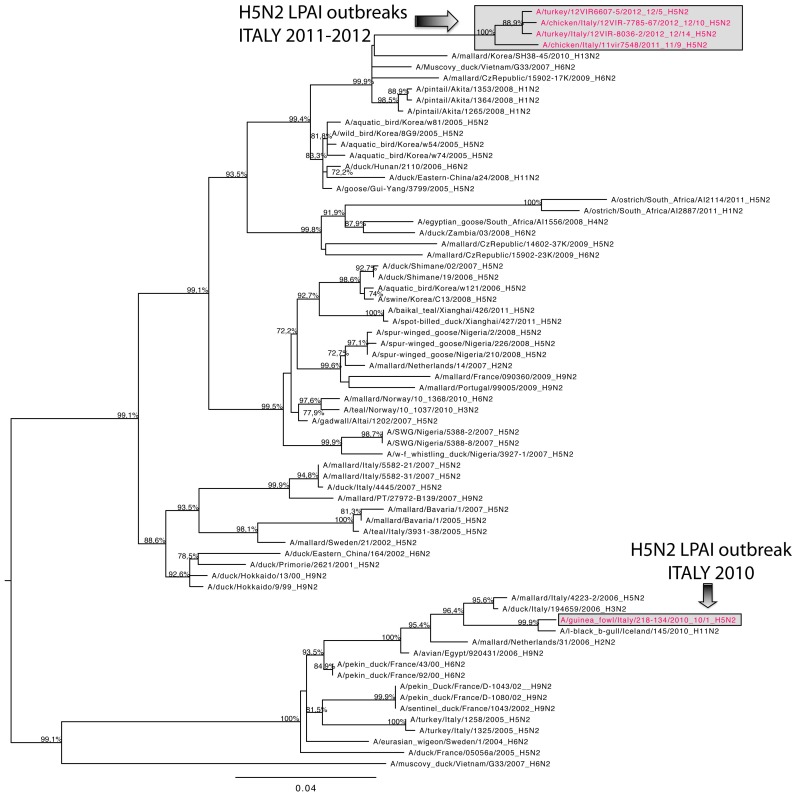
Maximum-likelihood phylogenetic tree for the neuraminidase gene segment of of Italian 2010–2012 H5N2 LPAI viruses. Viruses sequenced and characterized in this study are in red. Numbers at the nodes represent bootstrap values.

To explore the whole-genome evolution of viruses obtained from the Italian H5N2 LPAI outbreaks, ML trees were inferred using virus sequences available from seven different outbreaks. The topology of the HA phylogeny indicated that the virus strains from outbreak 12/10 identified in Veneto in 2012 and those collected in the same year in Lombardy (12/5, 12/6, 12/8 and 12/14) clustered in the same branch of the phylogenetic tree showing a high percentage of similarity (99.7%–99.9%) with each other and with the strain isolated from outbreak 11/9 (99.0%–99.1% similarity), which occurred in Campania in 2011. An identity of 97.6%–98% was found with the strain isolated from outbreak 10/1 which occurred in Veneto in 2010. This strain had a higher similarity with the strain A/spur-winged goose/Nigeria/2/2008 (H5N2) (99%) (Figure 5). All the viruses analyzed had typical low pathogenicity motifs at the HA cleavage site. In this portion of the HA gene, all 2012 virus strains presented a substitution of a non-basic amino acid (PQRETR*G) with a basic amino acid (PQRKTR*G). Phylogenies inferred for the other seven genome segments showed the same pattern as that of the HA gene for the viruses collected from the 2012 outbreaks (12/5, 12/10 and 12/14): the three 2012 viruses clustered together showing high similarity (99.6%–100%) with one another and with the strain isolated from outbreak 11/9 (98.7%–99.4%). Differently, the strain isolated from outbreak 10/1 resulted to be genetically distant (92.3%–97.8%) from the 2011–2012 strains for the NA gene (Figure 6) and for all the internal genes, with the only exception of the PB1 gene.

Amino acid analysis of the 2011–2012 strains demonstrated that all these viruses were characterized by an additional glycosylation site (NDA to NDT) in the position 253 of the HA gene, a stalk deletion in the NA gene (position 65–83) and by an elongation of the NS gene due to a mutation on the first base of the codon that usually encodes a stop signal in position 231. This mutation is a LVT (lenght variation type) reported in the literature as LVT (+7), which so far has been found only in human and swine viruses, with the exceptions of avian H7N3, H4N8, and H13N2 virus strains [20].

## Discussion

This study aimed at identifying potential environmental correlates of H5N2 LPAI outbreak heterogeneity as to determine environmental conditions where control efforts may be targeted to specific poultry populations. In parallel, phylogenetic characteristics of some of the virus strains were also investigated.

Despite general awareness that the observed patterns of AI viruses may entail some underlying ecological structure, there is a notable paucity of studies that have investigated the environmental drivers of H5N2 AI virus emergence in domestic poultry, e.g. [25], [26]. It is clear that ecological variation and disease occurrence are often mediated by complex, large-scale processes that are not immediately amenable to traditional approaches to causal inference [27]. Yet, a comprehensive approach assembling methodologies from diverse disciplines, including ecology, epidemiology, genetics and biogeography has been proposed to explore the spatio-temporal distribution of AI viruses through the combined application of statistical techniques from multivariate data analysis (ordination and clustering methods) and classical regression modelling [15]. These techniques are well suited to frame the disease ecology perspective using, for instance, data obtained from ongoing surveillance activities, allowing the findings to be informed by hypotheses about how ecological interactions among poultry populations, viruses and their environments can be related to the observed pattern of disease emergence [15].

Several environmental variables were dropped to build the final regression model because of their “weak” association with the outbreak ordination configuration. This allowed us to discern which of the considered variables could be deemed as robust and significant predictors of outbreak heterogeneity. The hypotheses behind the possible association of the considered environmental variables with outbreak heterogeneity varied according to the variable in question. For instance, it was hypothesized that larger human population densities around the infected premises would increase the odds of interactions between poultry and people, as well as the chance of viruses being transferred elsewhere, so that outbreak occurrence would be more heterogeneous (and so less predictable) over time, space and susceptible poultry populations. This also applies to those land cover classes specifying urbanized or infrastructured environments, as these would promote increased movements of human beings and commodities, including poultry and poultry products, which could favor long-distance viral spread to varying poultry populations. It was therefore not surprising to find that increased amount of land covered by industrial, commercial and transport units was significantly associated with increased outbreak heterogeneity. Conversely, the amount of arable land was negatively associated with outbreak differentiation. Indeed increased rural or agricultural areas may plausibly reduce outbreak heterogeneity, as both the low human density and the less developed infrastructural system usually present in the countryside would create an unfavorable condition for extensive viral circulation outside a given timeframe, geographical area or poultry production sector. Consequently, in such circumstances outbreaks would tend to be more (ecologically) similar to each other, involving only specific poultry populations and being temporally and spatially closer to one another.

Similarly to the hypothesized effect of human density, the density of susceptible hosts would influence outbreak differentiation via increased chance of viral circulation outside a given ecological context. Interestingly, outbreak heterogeneity was positively associated with the density of turkey and broiler breeder populations, but as the density of duck and goose farms and the density of poultry populations other than TLBDG increased, then outbreak heterogeneity decreased. While there is no apparent explanation for these two latter negative associations, it is true that poultry breeders are usually characterized by a longer productive lifespan than their fattening counterparts. Therefore, their likely exposure to, and amplification of, AI viruses might be more prolonged, possibly resulting in more extensive viral circulation and so increased unpredictability of outbreak occurrence. This speculation is supported, to a certain extent, by similar findings reported from Japan, where a high density of layer flocks containing “end-of-lay” hens (which usually have a relatively long productive lifespan of 1–2 years) was associated with the occurrence of 41 H5N2 LPAI outbreaks in 2005 [26], [25]. Yet, while the Japanese outbreaks also affected layers, breeder flocks were not affected in any of the Italian outbreaks. Thus it seems that outbreak heterogeneity is somehow only related to the poultry circuits at the top of the production chain and not to their longer production cycles per se.

Besides high poultry and human density areas, other important areas where increased viral circulation may take place are wetlands and water surfaces because of their usually high densities of (migratory) waterfowls, as well as the increased chance for surface waters in such circumstances to act as a vehicle for fecal-oral transmission of AI viruses [28]. Also the elevation can influence the risk for a poultry farm to become infected with AI viruses, with farms located at more than 150 m above sea level in Italy being at significantly lower risk than those located at lower altitudes [29], possibly because of increased geographical isolation and the presence of unrecognized local environmental conditions that make such areas less prone to experiencing AI outbreaks. Despite this hypothesized effect, our variables specifying surface water sites, altitude and waterfowl population around the infected flocks were not significant in the final regression model, although some of them exhibited a certain degree of correlation with the outbreak ordination configuration.

Flock type was the main determining factor of cluster assignment, suggesting that commonalities of flocks in the same poultry production circuits may overcome geography, time and type/amount of susceptible hosts in modulating the epidemiological similarities of the outbreaks. Studying associations between the environmental variables and the subdivision of outbreaks into clusters indicated that in areas where industrial, commercial and transport units were widespread, outbreaks were significantly more likely to affect industrial flocks of turkeys and layers nationwide. This also applied to areas with high densities of broiler and turkey breeders. In contrast, lowly infrastructured areas were significantly more likely to experience outbreaks in ornamental, commercial and rural multi-species flocks in the northern part of the country, while rural layer flocks in south-central Italy were significantly more prone to infection in areas with low densities of broiler and turkey breeders. Finally, areas with increased amounts of land covered by scrub and/or herbaceous vegetation (savannah-like environments) were significantly more likely to experience outbreaks in commercial flocks of galliformes, and less likely in industrial flocks, all over the country. Although it is not entirely clear as to how these environmental variables may be associated with the occurrence of H5N2 LPAI outbreaks in these specific ecological contexts, they may prove useful in categorizing several site-specific conditions where AI control efforts may be targeted, enhancing any intervention by matching a specific strategy rather than applying across-the-board methods. This seems to be applicable as in Italy most of the poultry industry has been concentrated in specific areas, where the high density of varying poultry holdings, hatcheries, abattoirs, feed mills, litter processing plants and other establishments is convenient from an organizational point of view, but it may pose a series of drawbacks in terms of increased risk of AI viruses spread [30]. Moreover, certain locations in Italy possess a more pronounced vocation to specific poultry productions than others. For instance, the Veneto region (particularly the province of Verona) is characterized by a high density of turkeys, while laying hens for table eggs are far more concentrated in Lombardy region. These are characteristics which may be framed in the specific environmental background to identify potential targets for control activities.

Despite the low number of available sequences, the results of the phylogenetic analyses substantiate, to some extent, those of the clustering based on the ecological characteristics of the outbreaks. For instance, the virus strains from outbreaks 12/10, 12/5, 12/6, 12/8 and 12/14 showed very high genetic similarity with one another and common molecular signatures were also identified in their HA, NS and NA genes. This was also reflected in the ecological clustering, as all these outbreaks clustered in two clusters (3 and 4) close to each other in ordination space (Figure 2). While this is suggestive of similar ecological niches for these virus strains, the limited number of sequences made it difficult to determine whether or not common environmental drivers were shaping the genetic commonalities identified among the viruses. Moreover, other likely determinants, such as the epidemiological connections among the outbreaks, may have played a major role as well. A larger array of sequences is needed to compare the evolutionary patterns (including rates of nucleotide substitution and selection pressures) of the viruses involved in different outbreaks in order to link the virus evolutionary properties to ecological factors.

Besides the lack of detailed genetic data for all the outbreaks as to examine to which extent genetic differentiation corresponds to ecological structuring, this study has some evident limitations due to the relatively low number of outbreaks which the analysis is based upon and the risk of falling into the trap of “ecological fallacy” in pursuing such a correlative analytical approach. Nevertheless, this study was based on a comprehensive set of fine-scale spatial data that allowed us to identify several environmental correlates of H5N2 LPAI outbreak heterogeneity. To the authors’ best knowledge, this is the first time that such analysis is applied to this kind of data.

As a final point, it is worth mentioning that the buffer radius of 10 km was chosen because it appears to be the average threshold for geographical spread of AI viruses between domestic poultry farms, i.e. where most of the viral transmission takes place as revealed by the clustering of infected farms [9] and the risk of transmission from infected to uninfected farms as a function of the inter-farm distance [31]. However, sensitivity analysis of the effect of the choice of the buffer radius on the results was also performed (data not shown). This was done by repeating the analysis with buffers at 1, 5, 15 and 20 km. As the results did not change significantly from one buffer to another, no further results were presented.

## Conclusions

Although it was not possible to infer any direct relationship of causation, results indicated that the pattern of (dis)similarities among the Italian H5N2 LPAI outbreaks entailed an underlying structure that may be the outcome of possible large-scale, environmental interactions in ecological dimension. Among the numerous environmental variables tested we identified increased population densities of broiler and turkey breeders, and increased amount of land devoted to industrial, commercial and transport units as possible ecological determinants of outbreak heterogeneity in time, space and susceptible poultry populations. Several environmental conditions associated with increased proneness of specific poultry populations to experiencing H5N2 LPAI outbreaks were also identified. These were conditions related to infrastructured and savannah-like environments and areas densely populated by poultry breeders. Although analytic integration of ecology and genetics was not possible due to the limited number of available sequences, suggestive evidence that ecological ordination makes sense genetically was provided, as virus strains showing high genetic similarity and common molecular signatures clustered into ecologically similar outbreaks. These results are in good agreement with current (eco)epidemiological knowledge on LPAI infection in Italy as derived from more traditional analytical approaches [9], [11], [32]–[34].

Considering outbreak heterogeneity rather than treating all LPAI outbreaks equally is important given the potential of these viruses to spread, evolve and increase pathogenicity. A better understanding of how ecological interactions among poultry populations, viruses and their environments can be related to the observed patterns of AI outbreaks may eventually enhance future interventions by implementing site-specific, ecologically-grounded strategies.
